# Natural competence in the bacterial pathogen *Xylella fastidiosa* varies across genotypes and is associated with adhesins

**DOI:** 10.1371/journal.ppat.1013757

**Published:** 2025-12-08

**Authors:** Ranlin Liu, María Pilar Velasco-Amo, Luis F. Arias-Giraldo, Monica A. Donegan, Neha Potnis, Nate B. Hardy, Rodrigo P. P. Almeida, Blanca B. Landa, Leonardo De La Fuente

**Affiliations:** 1 Department of Entomology and Plant Pathology, Auburn University, Auburn, Alabama, United States of America; 2 Institute for Sustainable Agriculture, Consejo Superior de Investigaciones Científicas (CSIC), Córdoba, Spain; 3 Department of Environmental Science, Policy and Management, University of California, Berkeley, California, United States of America; Ghazi University, PAKISTAN

## Abstract

Natural competence is one of the mechanisms of horizontal gene transfer, an important process that contributes to host-use evolution and other types of environmental adaptation in bacteria. Recently, the plant pathogen *Xylella fastidiosa* has undergone expansion of its host and geographic ranges. Natural competence has been empirically documented for a few strains of *X. fastidiosa*, but its prevalence across genotypes and populations is largely unknown. In this study, we characterized the natural competence in vitro of 142 *X. fastidiosa* strains from diverse hosts and geographic origins, and revealed substantial variability among strains, particularly across subspecies. *X. fastidiosa* subsp. *fastidiosa* strains were largely naturally competent, while only 15% of studied subsp. *multiplex* strains showed recombination, and none of the strains classified in other subspecies were competent. While recombination rates in vitro were associated with subspecies classification, host and climatic variables from the area of isolation did not explain differences in recombination across strains. A genome-wide association study identified several genes linked to variation in natural competence, including a heretofore unknown role for *xadA2*, which codes for a surface afimbrial adhesin, and the already known fimbrial adhesin type IV pili genes *pilY1-1* and *pilY1-3*. Overall, this study highlights the variability of natural competence among *X. fastidiosa* strains, that could have an impact on their potential for adaptation to the environment.

## Author summary

The pathogenic plant bacterium *Xylella fastidiosa* causes devastating diseases in the Americas in hundreds of crops including grapes, almonds and blueberries. In the last decade it was detected in Europe, where it is decimating olive trees in the south of Italy, among other plant hosts. Natural competence is a property of successful human and plant bacterial pathogens, that enables them to acquire genetic material from the environment and incorporate it in their genome. In theory, this allows these pathogens to adapt to new conditions faster and more efficiently by acquiring new properties. While its genetic basis has been characterized in some animal pathogenic bacteria, it remains less explored in plant pathogens where only a couple of species are known to be naturally competent. In this study, we revealed extensive variation in recombination rates within genotypes of the naturally competent *X. fastidiosa*. We identified for the first time the adhesin protein XadA2 as a key genetic factor associated with natural competence. Elucidating these mechanisms in a globally important plant pathogen not only advances our understanding of its evolution and adaptation but also offers broader insights into natural competence as a mechanism used by aggressive bacterial pathogens.

## Introduction

A distinguishing characteristic of some bacteria is their ability to naturally uptake DNA from their environment and integrate it into their chromosome through homologous recombination (HR). This process, known as natural competence, is one of the mechanisms of horizontal gene transfer, along with conjugation and transduction [1]. The DNA uptake process relies on the attachment of environmental DNA (eDNA) to FimT3 [2] and retraction of Type IV pilus (TIVP), which also mediates twitching motility [3,4]. After eDNA is brought into the periplasm by TIVP, it enters the cytoplasm where it can recombine with the chromosome through HR [5,6]. The whole process is tightly regulated and influenced by environmental and physiological factors such as genomic DNA damage, cell-cell communications, starvation, and certain carbon sources [7,8].

Although the ‘competence’ domain found in the key DNA-uptake processing gene *comEC* has been found in 96% of bacterial genomes, competence has been confirmed experimentally in < 1% of bacterial strains and predominantly observed in human pathogens [9,10]. *Xylella fastidiosa* is one of the two known naturally competent plant pathogens, alongside *Ralstonia solanacearum* [11–13]. *X. fastidiosa*, an insect-transmitted and xylem-limited plant pathogen, has a broad host range, infecting 712 plant species from 312 genera and 89 families [14]. Genotype classification includes three subspecies: *fastidiosa*, *multiplex*, and *pauca,* each with a proposed host range and putative geographic origin [15,16]. Additionally, two other subspecies names have been proposed and occur in the literature, subspp. *sandyi* and *morus*, although they are not phylogenetically supported [17–20]. In terms of host plants of economic importance, subsp. *fastidiosa* infects grape and almond; subsp. *multiplex* infects blueberry, almond, as well as a variety of weeds, shrubs and trees; and subsp. *pauca* infects olive, citrus, and coffee [14,20], among other hosts. Most subspecies have expanded their geographic ranges from their putative ancestral origins in recent decades.

*X. fastidiosa* is considered an emerging pathogen, with its introduction to Europe leading to novel plant-pathogen genotype associations [15,21,22]. Extensive HR, especially inter- and intra- subspecific HR, has been detected throughout genomes of *X. fastidiosa* strains and it is believed to be a primary force for genetic diversification [15,22,23]. Many recombination hotspots have been found in genes critical to the ecology of *X. fastidiosa*, suggesting the possible importance of HR for its adaptation and evolution [22]. Yet, the role of HR on *X. fastidiosa* adaptation remains inconclusive [24]. Natural competence (referring to the combined process of eDNA uptake followed by HR) in *X. fastidiosa* has been shown to contribute to HR in genomes under laboratory conditions [22,25], and variation on recombination rate via natural competence among *X. fastidiosa* was found on 13 strains isolated from the US [26]. Nevertheless, how recombination rates via natural competence varies across *X. fastidiosa* strains, and the genetic factors associated with such variation remain poorly understood. Here we characterized natural competence in 142 *X. fastidiosa* strains in vitro. We evaluated correlations between natural competence and various environmental factors, twitching motility, and 11 TIVP genes. Finally, utilizing a Genome-Wide Association Study (GWAS), we found a nonfimbrial adhesin gene associated with natural competence, whose novel role in this process was confirmed by mutagenesis analysis.

## Materials and methods

### Bacterial strains, plasmids, and culture conditions

Metadata information on *X. fastidiosa* wild-type strains, mutant strains and plasmids used in this study are provided in S1 and S2 Tables, respectively. Primers used to generate mutants are listed in S5 Table. Mutants were constructed using site-directed mutagenesis, following previously described methods [27]. All strains were grown on Periwinkle Wilt (PW) [28] agar plates modified by omitting phenol red and using 1.8 g l^-1^ of bovine serum albumin (BSA) (Gibco Life Sciences Technology) at 28°C for seven days, and re-streaked onto new PW plates for another week before setting up experiments. Natural competence assays were performed using PD3 medium [28] that was used to suspend cells in liquid and coculture cells with donor DNA. Luria Bertani (LB) liquid medium was used to culture *Escherichia coli* cells for extracting plasmids. Kanamycin (Km) and chloramphenicol (Cm) were used when needed at concentrations of 30 µg ml^-1^ and 10 µg ml^-1^, respectively.

### Genomes sequencing, assembling, and annotation

Eleven *X. fastidiosa* strains were newly sequenced using Illumina MiSeq at SeqCenter (Helomics Co., Pittsburgh, PA, US). Genomic DNA was extracted using a modified cetyltrimethylammonium bromide (CTAB) method [29]. All raw reads can be retrieved from NCBI BioProject accession number PRJNA1189278. Paired-end FASTQ reads were quality-assessed with FastQC v0.10.1 [30] and trimmed with BBMap v37.36 (Bushnell B. - sourceforge.net/projects/bbmap/) with default parameters to filter low quality reads and adapters. The trimmed reads were de novo assembled using SPAdes v3.15 [31] with the *careful* option turned on and *k*-values of 21, 33, 55, 77, 99, and 127. Assemblies were annotated using Prokka v1.13 [32]. Assembly quality was evaluated using QUAST v5.2.0 [33]. Additionally, for eight other strains (XYL2014/18, XYL2017/18, XYL2093/18, XYL2153/18, CFBP7969, CFBP7970, CFBP8082, and IAS-AXF64H11), DNA sequencing and *de novo* hybrid assembly were performed as previously described [34]. Genomic DNA was extracted using the Quick DNA Fungal/Bacteria Miniprep kit (Zymo Research Group). Libraries for Oxford Nanopore Technologies (ONT) sequencing were prepared by multiplexing, using the ligation sequencing gDNA and Native barcoding kit SQKNBD114.24 or the VolTRAX Multiplex Kit VMK004 in the VolTRAX v0.21.0 system, and sequenced on an R9.4 flow cell with the MinION device. Illumina sequencing libraries were prepared for IAS-AXF64H11 with the Illumina HiSeq 4000 platform. For the remaining strains, Illumina data were retrieved from the Sequence Read Archive (SRA) database: Strains XYL2014/18 (SRR11931330), XYL2017/18 (SRR11931328), XYL2093/18 (SRR11931329), XYL2153/18 (SRR11931325), CFBP7969 (SRR8454392), CFBP7970 (SRR8454394), CFBP8082 (SRR8454532). Raw long reads from ONT sequencing were trimmed using Porechop v0.2.4 [35]. *De novo* hybrid genome assemblies were generated by integrating Unicycle [36] with Canu [37]. Coding genes were predicted using the NCBI Prokaryotic Genome Annotation Pipeline (PGAP) v6.6 [38,39].

### Empirical measures of recombination via natural competence

Recombination rates via natural competence were assessed in 142 *X. fastidiosa* strains using donor DNA from plasmid pKLN61, which carries a Km resistance cassette inserted into *rpfF* of the Temecula1 strain [40]. The *rpfF* gene in *X. fastidiosa* encodes the synthase for the quorum sensing molecule Diffusible Signal Factor (DSF), mediating cell-cell signaling and regulating key pathogenicity traits and vector transmission [40]. This plasmid was chosen as it exhibited a higher recombination rate than other plasmids tested in a previous study [26]. Consistent with this, *rpfF* has been reported as a recombination hotspot [22]. Prior to transformation, the plasmid was methylated by a methylase derived from *X. fastidiosa* (PD1607) using the *E. coli* strain EAM1 [41]. Natural competence was further validated in a subset of strains using total DNA released from heat-killed *X. fastidiosa* donors harboring an antibiotic cassette inserted into a neutral site, which does not impact growth or virulence *in vitro* or *in planta* [42], following previous methods [26]. Briefly, *X. fastidiosa* strains were co-cultured with donor DNA or dead cells on PD3 plates for 5–7 days depending on the growth rate of specific strains. Cells were heat-killed by incubating cell suspensions (OD_600_ = 0.8) at 90 °C for 15 min and viability of cells were examined by spread plating 100 µL cell suspensions on PW plates. Recombinants were selected on PW plates with antibiotic selection according to the marker used. Recombination rate was calculated as the ratio of number of recombinants to total viable cells. To determine whether the comparatively lower sequence identity to subsp. *multiplex* strains contributed to their non-competency, we constructed a new plasmid donor DNA, pKLN61-A, carrying homologous arms of *rpfF* gene cloned from subsp. *multiplex* AlmaEm3 following a previously described method [40]. We obtained higher identity (99.93% compared to 97.58%) to subsp. *multiplex* strains that were used to test for recombination by natural competence (S4 Table). The plasmid pAX1-Cm [42] was used for natural competence assays for PD0744 mutant (Km^R^) and wildtype TemeculaL. Approximate sampling locations of strains and their recombination rates were visualized in R using ggplot2 [43]. The base map layer (country and regional borders) comes from the maps package, which provides openly available map data derived from the CIA World DataBank II (https://www.evl.uic.edu/pape/data/WDB/).

### Twitching motility assay

Twitching motility of *X. fastidiosa* strains was measured as described previously [25]. Briefly, cells of each strain were scraped from PW plates using a sterile loop and then spotted on PW plates without BSA using a toothpick. After four days of incubation at 28°C, colony peripheral fringe of each spot was observed under 10X magnification using a Nikon Eclipse Ti Inverted Microscope (NIKON, Melville, NY), and photos of six colonies per plate per strain were taken for measurements of fringe width using ImageJ software [44].

### Phylogenetic analysis

The scripts and parameters used in all the bioinformatic analyses are accessible at Github repository: https://github.com/dlf-xyl/natural-competence. Genome assemblies of 133 *X. fastidiosa* strains were obtained from NCBI or sequenced here. A core-genome alignment was obtained using Roary v3.13.0 [45]. The core-genome Maximum Likelihood (ML) phylogenetic tree without removing recombination regions was estimated using RAxML v8.0.24 [46] with the GTR + Γ model (generalized time-reversible model with Γ-distributed among-site rate heterogeneity) and 1,000 bootstrap replications. Mid-point rooted ML trees along with metadata were visualized in iToL [47]. The host-use of each strain was expressed at the genus level, except for host species with only a few strains, which were classified as using hosts of class “others.” This group includes the following species: *Rhamnus alaternus*, *Cercis* spp., *Ambrosia* spp., *Polygala myrtifolia*, *Ficus carica*, *Santolina chamaecyparissus*, *Carya illinoinensis*, *Quercus* spp., *Lupinus* spp., *Platanus occidentalis*, *Helianthus annuus*, and *Iva angustifolia.*

### Modeling analysis

To decompose the variation in recombination rates across putative environmental and genomic predictors, we used generalized linear mixed-effect models fit with the *bmrs* package in R [48]. The model assumes a log-normal hurdle distribution for response variables to account for the skewed distribution (S3A Fig) of the response variable, *ν*, which is the estimated recombination rate via natural competence. The comparative dataset comprises 281 measures of recombination rate, sampled across 133 strains. To account for the non-independence of our observations of *ν* due to shared ancestry, the model included a random intercept based on a phylogenetic covariance matrix. The matrix was derived from a midpoint-rooted phylogeny and assuming Brownian motion. To account for the potential dependence of *ν* on the host environment in which a *X. fastidiosa* strain was isolated, the host plant genus from which it was sampled was also included as a random effect. We also included a random effect of binned sampling location (i.e., Europe, Central America, South America, Southeastern US, Western US) to account for geographic sampling bias. To assess the potential relationship between recombination rate phenotypes and the environment, we included as fixed effects three indices of climate variation: the first three components derived from a principal components analysis of the 19 so-called bioclimatic variables from WorldClim database v2.1 [49,50]. All 19 numeric bioclimatic variables were centered and scaled prior to the PCA. These three principal components account for 82.6% of the climate variance across strain sampling locations (S3B and S3C Fig). Loadings on the first component (40.2% of variation) indicate that it primarily captures variation in total annual precipitation (bio12). The second component (26.4%) captures temperatures in the coldest month (bio6) and coldest quarter (bio11), whereas the third (16%) captures temperatures in the warmest quarter (bio10) (S3B Fig). To fit the phylogenetic regression model, we chose informative priors to help with model convergence. Both the intercept (i) and population-level effects (β) were parameterized with a normal distribution of i~N(0,10) and β~N(0,5), while the standard deviation (sd) and residual standard deviation (sigma; σ) had student’s t-distribution priors of sd~student(3,0,20) and σ~student(3,0,20).

### Comparative genomic and gene content analysis

To assess if differences in TIVP genes explained differences in recombination rate via natural competence in vitro, we focused on genes that were shown previously to influence natural competence in *X. fastidiosa* [2]. Amino acid sequences of the 11 TIVP genes selected in *X. fastidiosa* were obtained using automlsa2 (v0.7.1; https://github.com/davised/automlsa2), which utilized the sequences from Temecula1 as queries for BLASTP to retrieve homologs with 30% coverage and 50% identity cutoff. Automlsa2 used MAFFT [51] to generate amino acid sequences alignment. The phylogeny, along with sequence percentage identity and alignment was visualized using iTOL [47]. Prophage regions of Temecula1 were detected with Prophage Hunter [52].

### Genome-wide association study

To identify genetic factors associated with natural competence, genome assemblies of 133 strains that were characterized for natural competence in vitro were analyzed further. Genomes from 9 strains out of the 142 strains tested in vitro were not available, therefore only 133 strains were used for this analysis. The natural competence phenotype was expressed as a binary or ordinal (0–3, from no recombinant to highly recombinant) variable (S6 Table). For the sake of robustness, we used three different tools, i.e., treeWAS [61], Pyseer v1.3.10 [53], and DBGWAS v0.5.4 [54] with default settings. The program treeWAS utilized a phylogenetic tree-based approach that accounts for recombination and clonal population structure. Recombination was not removed from genomes during this analysis. treeWAS was performed using SNPs and phylogeny of the 133 strains generated by ParSNP [55] which used Temecula1 as the reference genome. Pyseer and DBGWAS use *k*-mer-based approach which relies on linear mixed models; for these analyses we used the core-genome phylogeny re-constructed as described above to account for population structure. Significant *k*-mers identified by DBGWAS were mapped to the protein database of *X. fastidiosa* subsp. *pauca* strain 9a5c downloaded from UniProt database [56]. Significant *k*-mers were mapped to three reference genomes of representative *X. fastidiosa* strain from three subspecies: Temecula1 (subsp. *fastidiosa*), AlmaEm3 (subsp. *multiplex*), and 9a5c (subsp. *pauca*) and annotated by Pyseer annotation pipeline. Quantile-quantile (QQ) plots were generated by Pyseer.

### Statistical analysis

Twitching motility of 35 *X. fastidiosa* strains were analyzed using two-tailed Student’s *t*-test to compare tested strains with the model strain TemeculaL. To examine the correlation between natural competence and twitching motility, we calculated pairwise Pearson correlation coefficients between the recombination rates of natural competence and the log-transformed twitching motility. Additionally, we employed linear mixed-effects models, incorporating subspecies as a random effect, using the lme4 package in R [48].

## Results

### Extensive variation in natural competence among *X. fastidiosa* strains

Among the 142 strains tested in vitro from various hosts and geographic locations, 51% were naturally competent in vitro, while 49% were non-competent (S1 Fig and S1 Table). Here, we classify as non-competent those strains for which we observed no evidence of recombination of the antibiotic resistance cassette included in the plasmid used as donor DNA. However, it is possible that such strains could internalize DNA via natural competence and recombine, but at rates too low for us to detect under the conditions of our assay. Natural competence appeared to be associated with subspecies classification: 98% of subsp. *fastidiosa* strains were naturally competent, whereas only 15% of subsp. *multiplex* strains showed competency in vitro. Due to the limited number of strains from subsp. *pauca* (6 strains), subsp. *sandyi* (7 strains), and subsp. *morus* (5 strains), we cannot rule out that strains in these subspecies are naturally competent. The recombination rates among the 142 strains ranged from 3.17 × 10^-8^ (Riv19, subsp. *morus*, isolated from mulberry in CA, USA) to 4.14 × 10^-1^ (16M3, subsp. *fastidiosa*, isolated from grape in GA, USA). To rule out a marker effect specific to *rpfF* gene, we tested natural competence again in seven non-competent strains from subsp. *multiplex* and *pauca* using genomic DNA from heat-killed subsp. *fastidiosa* and *multiplex* strains as donor DNA (S2 Table). None of these strains recombined with these donor’s DNA which harbors antibiotic markers at the neutral site (S3 Table). To assess whether sequence identity of homologous region flanking *rpfF* gene in the donor DNA affected natural competence, we compared sequence identity between donor DNA and recipient genomes of 48 strains (including 15 non-competent strains) using BLAST. Flanking region identities averaged 100% for subsp. *fastidiosa* strains, 97.6% for subsp. *multiplex* and 96.2% for subsp. *pauca*, with the lowest being 93.4% in subsp. *pauca* strain De Donno (S4 Table). To further assess whether the sequence identity affects natural competence, we tested natural competence in 11 non-competent subsp. *multiplex* strains using plasmid pKLN61-A, which showed 99.9% sequence identity with subsp. *multiplex* strains. However, no recombinants were obtained with this plasmid whereas the reference competent strains TemeculaL and AlmaEm3 successfully generated recombinants (S3 Table). Additionally, we tested subsp. *pauca* De Donno with donor DNA fragments containing identical flanking regions of three different TIVP genes cloned from its own genome using primers listed in S5 Table, but no recombinants were recovered (S3 Table). The non-competency of De Donno is consistent with previous work [57]. These results indicate that the donor DNA we used probably does not account for the variation in natural competence across subspecies.

**Fig 1:**
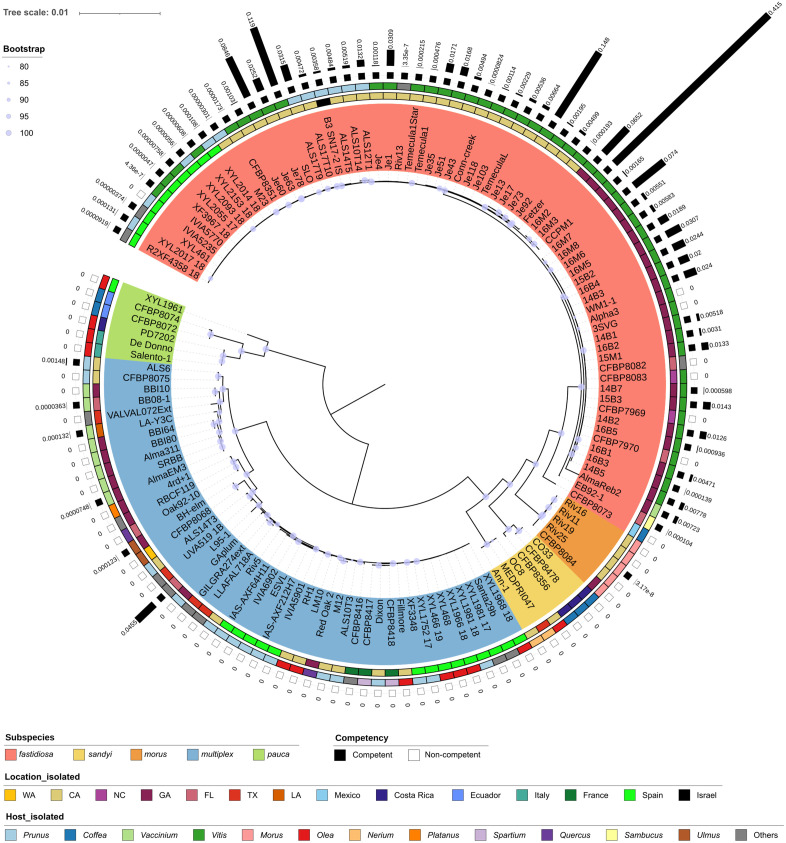
Midpoint-rooted maximum likelihood phylogenetic tree of 133 *X. fastidiosa* strains based on core genome alignment. Strain name background colors correspond to the subspecific classification. The inner ring of annotations denotes the host genus of isolation, with some underrepresented genera classified as “others.” The middle ring indicates the geographic location of isolation, and the outer ring shows the rate of natural competency and recombination in vitro. The black bars represent mean values of recombination rate, where “0” means the recombination rate is below the detection limit. Bootstrap confidence values were calculated from 1,000 replicates, and values above 80 are shown on the tree. Phylogenetic distances are represented by a branch length of 0.01 substitutions per site. The tree was visualized using iTOL [47].

**Fig 2:**
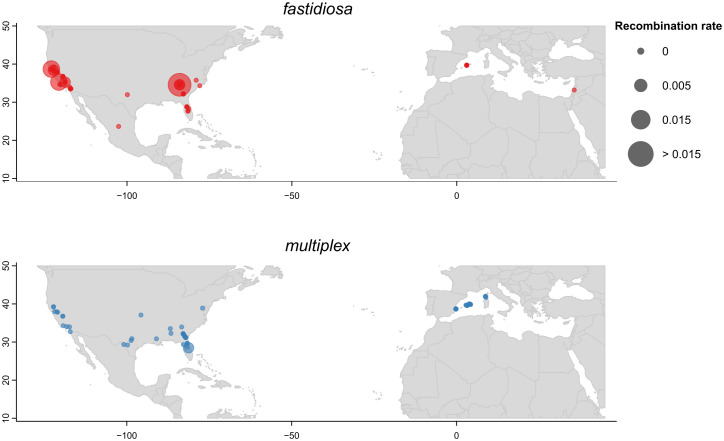
Global distribution of location of isolation of *X. fastidiosa* subsp. *fastidiosa* and subsp. *multiplex* strains tested in this study, grouped by subspecies. The dot size indicates values of recombination rates. Other subspecies are included in S2 Fig. The map was created using the R package ggplot2 [43] and maps. The base map layer (country and regional borders) comes from the maps package, which provides openly available map data derived from the CIA World DataBank II (https://www.evl.uic.edu/pape/data/WDB/).

Genome assemblies were available for 121 of 142 tested strains. By combining these assemblies with previous results of an additional 12 strains [26], we constructed a core-genome phylogeny of 133 strains, the relationships of which were plotted alongside their host, geographic origins, and recombination rate by natural competence in vitro (Fig 1). Importantly, this phylogeny was generated without filtering for recombination events, which limits its resolution for certain clades —particularly for subsp. *morus* which clusters within subsp. *fastidiosa*, and subsp. *sandyi,* which appears as a basal lineage to subsp. *fastidiosa*. Nevertheless, the phylogeny indicates that natural competence is more associated with subspecies classification than with host or geographic location. For instance, strains isolated from almond include both subsp. *fastidiosa* and *multiplex* and shared similar geographic origins of California and Europe. But only those classified as subsp. *fastidiosa* (11 strains) were competent, while all 16 subsp. *multiplex* almond strains were non-competent, except for strain ALS6. Similarly for European strains, only subsp. *fastidiosa* strains were naturally competent, while most European strains from other subspecies were non-competent (S1 Fig). However, it is important to note that our dataset is biased due to the lack of diversity among strains collections worldwide, for instance subsp. *fastidiosa* strains used here had less diversity in host and geographic origins compared to other subspecies, with 75% isolated from grapevines and 72% isolated from California and Georgia in the US. The geographic distribution of all 154 strains tested in vitro (142 strains here plus 12 tested previously [26]) were plotted with their recombination rates (Figs 2 and S2). Both the map and phylogeny show that strains of subsp. *fastidiosa* with higher recombination rates are mainly from Georgia and California, while European subsp. *fastidiosa* strains tend to show lower recombination rates. Among the eight non-competent subsp. *fastidiosa* strains, five were from southeastern US in North Carolina, Georgia, and Florida. Notably, the non-competent subsp. *fastidiosa* strains were scattered throughout their respective subclades. In contrast, competent subsp. *multiplex* strains were primarily located on one of the two clades of subsp. *multiplex* (Fig 1).

### Environmental factors do not correlate with recombination rate

To explore if environmental factors play a role in shaping natural competence during evolutionary processes, we built a generalized linear mixed model, while accounting for host association, strain relatedness, and geographic provenance as random effects. None of the three environmental principal components showed significant effects on recombination rate (S3D Fig). For the three random effects, we examined by-group intraclass correlation coefficients (ICC), a metric of how much variance can be explained by the grouping within each random effect. Strain relatedness explained the most variance in recombination rate (ICC = 0.8), whereas host genus and location explained less (ICC = 0.043 and 0.152, respectively). Overall, differences in empirical recombination rate could be largely explained by phylogenetic signal, precluding any significant environmental effects in this model.

### Correlation between natural competence and twitching motility

**Fig 3:**
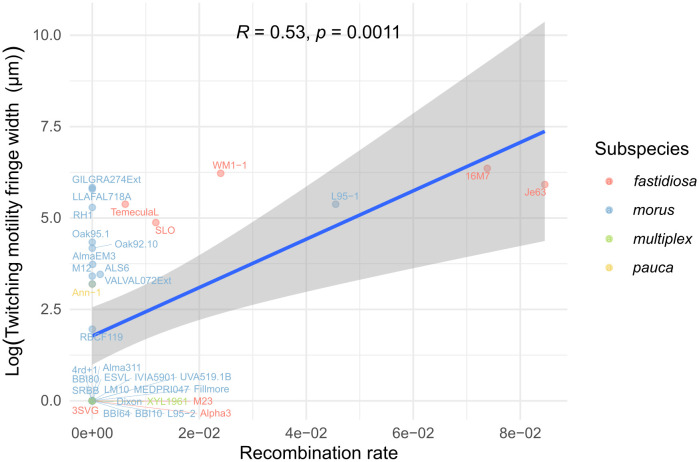
Correlation analysis between natural competence represented by recombination rate and twitching motility represented by log-transformed fringe width, color-coded by subspecies. Recombination rate was calculated as the ratio of number of recombinants to total viable cells. Pearson correlation was used to assess the linear relationship between natural competence and twitching motility.

To further investigate the genetic factors associated with natural competence, we first focused on the role of TIVP as it functions as the DNA uptake machinery in *X. fastidiosa* [2]. Given the essential role of TIVP in twitching motility and previous findings that twitching motility is positively correlated with natural competence in *X. fastidiosa* based on an analysis of 12 strains, we aimed to determine whether this correlation holds across a larger set of strains [26]. We measured the fringe width representing twitching motility of thirty-five strains, twenty-six of which were non-competent (S4 Fig). Consistent with previous findings, twitching motility was significantly positively correlated with natural competence (R = 0.53, *P* = 1.1e-3) (Fig 3). However, since subspecies differences may influence this relationship, we fitted a linear mixed-effects model incorporating subspecies as a random effect. This model revealed that *X. fastidiosa* subspecies account for a substantial proportion of variance (adjusted ICC = 0.495). Despite this, a significant positive relationship between twitching motility and natural competence remained (coefficient = 0.41; 95% CI = 0.28–0.56), indicating that the association persists even after controlling for subspecies-level variation. However, several outliers, mainly strains of the subsp. *multiplex*, displayed high twitching motility but lacked competency, while strain M23 was naturally competent despite no visible motility on agar plates.

**Fig 4:**
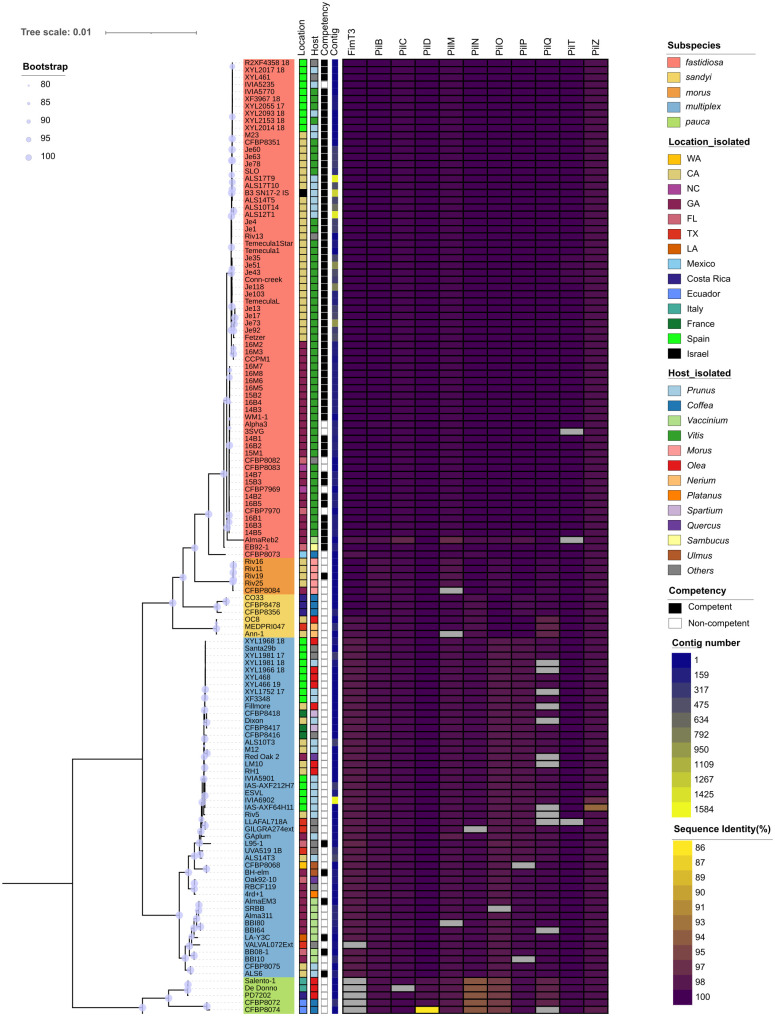
Comparison of percentage identity of 11 TIVP proteins across 133 *X. fastidiosa* strains. Strain background colors indicate subspecies classification. The columns, from left to right, denote geographic location of isolation, the host genera of isolation (with some underrepresented genera grouped under “others”), competency (indicated by solid squares for competent strains and empty rectangles for non-competent strains), contig numbers reflecting genome assembly completeness, and percentage identity of the 11 TIVP proteins. The grey color indicates mutation resulting in deletions of more than one amino acid within this protein. The mutation and percentage identity of amino acid sequences were identified by automlsa2 (v0.7.1; https://github.com/davised/automlsa2). The phylogeny was visualized using iTOL [47].

Previous work identified 10 TIVP genes (*pilB, pilC, pilD, pilM, pilN, pilO, pilP, pilQ, pilT, pilZ*) that are essential for both natural competence and twitching motility, and one DNA-binding protein (FimT3) essential for natural competence but not twitching motility in TemeculaL [2]. Among them, *pilM, pilN, pilO, pilP,* and *pilQ*, are in the same operon while *pilC*, *pilD*, *coaE* (PD1921) are in a different operon [2]. To evaluate sequence variation at the protein level, we used BLASTp to estimate the amino acid sequence divergence of the corresponding proteins across the 133 strains. We found that these proteins are generally conserved across *X. fastidiosa* strains, though some strains exhibited mutations in different TIVP proteins (Fig 4). Of the 11 TIVP proteins analyzed, PilB, PilD, and PilZ were the most conserved showing complete homologs across our dataset, though some strains still have some substitutions or deletions. PilQ was found to have more mutations than other analyzed proteins, with 12 strains from various origins displaying different types of mutations (S5A Fig). All tested 6 subsp. *pauca* strains displayed mutations in FimT3 (S5B Fig). In addition to mutations, subsp. *pauca* strains showed lower sequence identity in PilO and PilN among the 11 proteins. Notably, twenty-six non-competent strains primarily from subsp. *multiplex* and *pauca* displayed deletions of more than one amino acid in at least one of the tested proteins. By contrast, only one competent strain, AlmaReb2 (subsp. *fastidiosa*, isolated from blueberry in Georgia, US) exhibited such a deletion specifically in the PilT protein (S5C Fig). Of the 15 tested strains that are non-competent and non-motile, eight had mutations in multiple TIVP proteins. Among the eight tested strains that are motile but not competent, four showed mutations in TIVP proteins. Interestingly, no mutations were found in these 11 proteins for the competent but non-motile strain M23.

### Genome-wide association study

**Fig 5:**
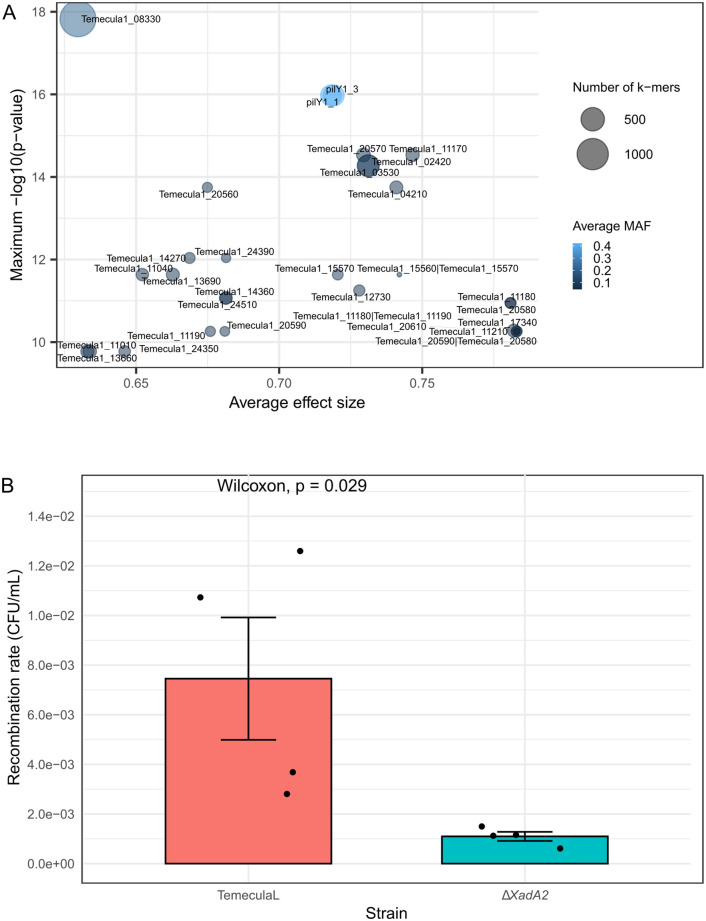
(A) GWAS results from Pyseer program to identify competence-associated genes. Values of maximum –log_10_(*P*) in the y axis were plotted against average effect size in the x axis. **(B)** Recombination rates of *X. fastidiosa* TemeculaL wildtype and *xadA2* (PD0744) mutant strain. Quantification of recombination rate were performed in PD3 plates by co-culturing cells with pAX1-Cm plasmid used as donor DNA and count the percentage of chloramphenicol-resistant (Cm^R^) recombinants in total viable population. Data represent means and standard errors of six independent replicates. Wilcoxon test was used for statistical comparison.

To investigate additional genetic factors associated with natural competence, we conducted a genome-wide association study (GWAS) on the 133 *X. fastidiosa* strains to identify statistically associated unitigs (*k*-mer sequences of certain lengths) or single nucleotide polymorphisms (SNPs). Using a binary natural competence phenotype measured in vitro, DBGWAS identified 130 significant unitigs out of a total of 2,585 unitigs, grouped into 30 subgraphs. In the top subgraph with minimum q-value, which had the lowest q-values (indicating the strongest association), 97% unitigs were mapped to *pilY1-1* (PD0023 in Temecula1) and *pilY1-3* (PD0502 in Temecula1) (S6A Fig). In the second top subgraph, all but 2 of 357 unitigs were mapped to gene *xadA2* (PD0744 in Temecula1) (S6B Fig). Notably, DBGWAS also identified several significant unitigs in these subgraphs that were negatively associated with natural competence (S7 Table), suggesting that presence of certain SNPs, indels or insertions in these unitigs may disrupt natural competence. Consistent with this, Pyseer identified the strongest associations between genes of *xadA2*, *pilY1-1* and *pilY1-3* (Fig 5A). When using an ordinal classification of recombination rate variation, with Pyseer, we identified additional candidate genes, including phage-related and hypothetical protein genes (S7 Fig). Quantile-Quantile (Q-Q) plots revealed no issues related to population structure regardless of the phenotypes used (S8 Fig). In contrast, treeWAS did not identify any significant SNPs associated with natural competence, regardless of how variation in natural competence was encoded (S9 Fig).

The genes *pilY1-1* and *pilY1-3* share nearly identical sequences, making it challenging to differentiate between them and thus identifying both as associated with natural competence. It is possible that one or both are associated with natural competence variation in *X. fastidiosa*. Due to the draft status of many assemblies, attempts to examine the copy number variation of *pilY1-1/pilY1-3* were unsuccessful, with some exhibiting a high number of contigs. To further investigate the effect of *xadA2* on natural competence, we constructed a PD0744 mutant in TemeculaL using site-directed mutagenesis. The *xadA2* mutant exhibited a significantly lower recombination rate compared to the wildtype (Fig 5B), suggesting it plays a role in natural competence. Interestingly, *xadA2* was identified within an inactive prophage region consisting of 16 genes in Temecula1 (S8 Table).

## Discussion

We uncovered significant variation in natural competence among *X. fastidiosa* strains. While a previous investigation of 13 *X. fastidiosa* strains isolated from the US reported that ~85% were naturally competent [26], our broader assessment found this to be true of only about half of 142 strains. This indicates that the ability to undergo natural competence in vitro may not be as widespread in *X. fastidiosa* populations as previously assumed. Recombination rates among naturally competent strains varied by seven orders of magnitude. This level of heterogeneity has also been observed in other species of naturally competent bacteria [11,58–64]. Phylogenetic analysis revealed distinct partitioning of natural competence across subspecies, with most subsp. *fastidiosa* strains being competent, while most subsp. *multiplex* strains were not. Notably, the only naturally competent subsp. *multiplex* almond strain, ALS6, forms a distinct phylogenetic group compared with other non-competent subsp. *multiplex* almond strains. This pattern indicates that ALS6 may have retained or gained genetic factors associated with natural competence, potentially through horizontal gene transfer or relaxed selective constraints, although further investigation is needed.

Previous studies have divided subsp. *multiplex* into two groups based on recombination in their genes (MLST analysis) or genomes (core-genome analysis): a group that does intersubspecific homologous recombination (IHR) and one that does not (the non-IHR group) [15,17]. Our empirical natural competence results partially align with this classification: competent subsp. *multiplex* strains tend to belong to the IHR group, while most strains from non-IHR group were not naturally competent, except for one strain, L95-1, which showed a relatively higher recombination rate than other competent subsp. *multiplex* strains. Notably, many closely related strains within the IHR group—despite originating from the same host and geographic regions as the competent strains—did not exhibit natural competence. Several sequenced strains from the IHR group and subspp. *pauca*, *sandyi*, and *morus,* previously reported to harbor abundant recombination events in their genomes [23,65], were also unexpectedly shown not to recombine in vitro. This discrepancy may reflect limitations of our experimental setting that may be unable to detect very low rates of recombination in vitro, or may lack specific, yet unidentified, environmental inducers to trigger natural competence in these strains. Alternatively, the recombination events observed in their genomes could be the result of other horizontal gene transfer mechanisms, such as conjugation or transduction, particularly in ecological contexts where diversifying selection favors and maintains genetic variation.

Our comparative analyses did not support a role for climate in shaping recombination rates. Our model points to a strong phylogenetic signal for this trait, which is logical as most of the competent strains were nested within subsp. *fastidiosa*. *X. fastidiosa* has a complex biogeography, and other factors–such as recent introductions or host shifts–may also impact whether high recombination rates are advantageous. According to reconstructions of historical biogeography, *X. fastidiosa* originated in the Americas and was only recently introduced to Europe [21,66,67]. Previous findings suggest that more frequent HR occurs in native populations compared to recently introduced populations [23,66]. Castillo et al. [24] proposed that HR might be adaptive by promoting the circulation of diverse alleles within older populations, while allele homogenization within more recent populations allows for efficient colonization of new host plants.

In line with previous research, our expanded analysis also reveals a positive association between twitching motility and natural competence in *X. fastidiosa* [2,26]. In contrast to previous research, we also identified a subset of non-competent strains that remained motile via twitching, with some showing deletions in amino acids among the 11 tested protein. While 10 of these proteins were selected for being essential for both natural competence and twitching motility in the *X. fastidiosa* TemeculaL strain [2], it appears that mutations in these essential TIVP proteins do not necessarily disrupt twitching motility in other genetic backgrounds. However, the expression levels of mutated proteins in different strains are yet to be assessed. In our analysis, 40% of non-competent strains displayed deletions of more than one amino acid in various TIVP proteins, which may account for their loss of competence. Only one of 68 (~1.5%) competent strains exhibited such deletions, consistent with a critical role of TIVP in natural competence. Remarkably, PilQ exhibited the highest number of deletions among the 11 proteins in our dataset, suggesting either that *pilQ* might be more prone to mutation resulting in premature stop codons or frameshifts, or that it is under diversifying selection across *X. fastidiosa* strains. PilQ is a multimeric outer membrane protein and plays a key role in DNA uptake during natural competence [68]. Similarly, in the naturally competent bacterium *Pseudomonas aeruginosa, pilQ* also shows a higher number of mutations among TIVP genes [69], and its mutation has been linked to phage resistance [70,71]. It is possible that mutations in PilQ in *X. fastidiosa* might confer an advantage by helping bacteria evade phage infections. Consistent with previous findings [2], we found evidence that mutations in FimT3 could contribute to loss of competence in subsp. *pauca* strains, although additional data from this subspecies are needed. In contrast, subsp. *multiplex* displayed mutations in different TIVP proteins, not just FimT3, implying that changes in TIVP proteins may vary across subspecies.

Our GWAS identified *xadA2* (PD0744) and *pilY1-1*/*pilY1-3* (PD0023/PD0502) as the genes most strongly associated with variation in natural competence across the strains of *X. fastidiosa* tested. Interestingly, both genes encode adhesins and have three paralogs or orthologs each. In *X. fastidiosa* strain Temecula1, there are three homologous genes encoded for tip adhesins: *pilY1-1, pilY1-2,* and *pilY1-3.* Previous studies have demonstrated that the deletion mutants ∆*pilY1-1* and ∆*pilY1-3* exhibited antagonist effect on natural competence [2]. Specifically, ∆*pilY1-1* lost its competency while ∆*pilY1-3* showed significantly higher natural competence rate compared to the WT [2]. *xadA2* encodes an outer membrane afimbrial adhesin with two additional orthologs, *xadA1* and *xadA3*, in the Temecula1 strain [72]. This finding supports the previously proposed idea that paralogs or orthologs in *X. fastidiosa* may have neofunctionalization [2]. Orthologs of *xadA* genes are present in the majority of complete or high-quality draft genomes of *X. fastidiosa* [73]. XadA2 plays a crucial role in biofilm formation and may inhibit cell-cell aggregation and twitching motility in *X. fastidiosa* [72,74,75]. Other surface proteins that impact both adhesion and natural competence have been also identified in other naturally competent bacteria [76–78]. It has been hypothesized that these proteins may influence natural competence by inducing surface modifications that facilitate DNA binding [76], which could also apply to XadA2 in *X. fastidiosa*, as its knockout enhances twitching motility [72]. Furthermore, RNA-Seq data from the *X. fastidiosa xadA2* mutant revealed a small change (34 genes) in expression profile in broth condition, suggesting *xadA2* that may influence natural competence indirectly [72]. A recent study [79] found that a *xadA2* mutant in Temecula1 exhibited altered oxylipin production and hypothesized that *xadA2* may play a role in activating the synthesis of oxylipin 7,10DiHOME, which could be a signal molecule in *X. fastidiosa*. This further implies that *xadA2* may regulate natural competence through complex regulatory pathways. *xadA2* was identified within an inactive prophage region in Temecula1, and several hypothetical phage-related genes were found to be significantly associated with natural competence when scaled competence phenotypes were used in GWAS. This suggests that prophage regions may influence natural competence, as seen in other naturally competent bacteria [80–82]. For example, prophage regions may become reactivated, targeting TIVP or other competence-related genes during infection, thereby influencing natural competence.

Although we have gained new insights into the causes of variation in natural competence in *X. fastidiosa*, they are contingent on certain methodological limitations, and some challenges remain. For instance, the limited and somewhat biased sampling of strains across subspecies, geographic locations, and hosts [83] used here restricts our ability to generalize from our data. Future studies should aim to test more strains from more diverse hosts and geographic regions. Additionally, our study tested natural competence only under standard batch culture conditions. Other conditions, such as microfluidic systems, plant environments, or alternative media, were not explored, though we did rule out donor DNA bias in our experimental setup. Similarly, twitching motility was only measured using agar plates. Alternative experimental conditions such as microfluidics or in planta assays could yield different patterns. The analysis of TIVP proteins revealed not only the deletions in proteins examined in this study but also the presence of numerous substitutions, insertion or frameshift, whose potential relationships with natural competence require further investigations. Additionally, future studies examining nucleotide diversity, selective pressure, and recombination-to-mutation rates in TIVP genes or proteins and other competence-related genes in *X. fastidiosa* populations, could provide further insights into the evolution of natural competence. While we found that *xadA2* is associated with natural competence in *X. fastidiosa*, further studies are needed to reveal its precise role. Investigations such as measuring its expression levels, protein structure, and function will provide deeper insights into its contribution to natural competence in *X. fastidiosa*. Differences in the number of types of Restriction Modification (R-M) systems and specificity subunits in Type I R-M system among *X. fastidiosa* subspecies have also been observed, with R-M systems known to be associated with natural competence in *X. fastidiosa* and other naturally competent bacteria [13,73,84–86]. This suggests that variation in natural competence may be linked to differences in R-M systems, warranting further investigation into their functional and evolutionary associations.

## Conclusions

Here, we have documented pronounced variation in the natural competence of strains of *X. fastidiosa* and identified genetic factors that correlate with that variation. Specifically, variation in natural competence appears to be linked to allelic variation in several TIVP proteins, and especially the afimbrial adhesin XadA2, possibly because of its roles as a regulator and in promoting DNA binding. The physiological mechanisms of natural competence are complex, and we have yet to work out many of the details.

## Supporting information

S1 Fig Recombination rate of 142 *X. fastidiosa* strains organized by subspecies classification. The recombination frequency was tested in vitro (see Materials and Methods) and data was log_10_-transformed. No bar indicates the recombination rate was below the detection limit and the strain was non-competent. Different bar colors indicate different subspecies. The black horizontal line indicates the average recombination rate of the reference strain TemeculaL. Experiments were repeated independently 1–3 times. Data represents means and standard errors.
(TIFF)

S2 Fig Global distribution of location of isolation of tested *X. fastidiosa* strains from subsp. *pauca*, *morus*, and *sandyi*. Subspecies are color-coded. The dot size indicates range of recombination rates. Subsp. *fastidiosa* and *multiplex* are shown in Fig 2. The map was created using the R package ggplot2 [43]and maps. The base map layer (country and regional borders) comes from the maps package, which provides openly available map data derived from the CIA World DataBank II (https://www.evl.uic.edu/pape/data/WDB/).
(TIFF)

S3 Fig Modeling and analysis of recombination data. (A) Histogram representing the distribution of recombination rates in log scale. The distribution is heavily skewed, with a large number (n = 65) of “0” values. The y-axis is disjointed to accommodate the skewed distribution. (B, C) Principal Component Analysis (PCA) biplot of climatic variables showing the relationship between principal component pairs PC1 and PC2 (B)/ PC2 and PC3 (C) and variable contributions. Arrows represent variables with the greatest contributions: bio6 (minimum temp. of the coldest month), bio11 (mean temp. of the coldest quarter), bio12 (annual precipitation), bio4 (temp. seasonality), bio1 (annual mean temp.), bio8 (mean temp. of the wettest quarter), and bio10 (mean temp. of the warmest quarter). The length and direction of the arrows reflect the strength and direction of their correlation with the principal components. Only the labels of the top variables are displayed for clarity in each plot. (D) Estimated fixed effects for covariates on recombination rate in a generalized linear mixed-effect model. Boxplots indicate the 95% credible interval; all fixed effects were not significant, as they were all overlapping with 1.
(TIFF)

S4 Fig Twitching motility of 35 *X. fastidiosa* strains. (A) Representative microscopic images of colony fringe of 35 tested *X. fastidiosa* strains grouped by subspecies. Images were captured at 10 × magnification. The scale bar on the first panel indicates 100 μm. (B) Quantitative twitching motility of the 35 *X. fastidiosa* strains, color-coded by subspecies. Twitching motility was determined by measuring fringe width of colonies spotted on PW plates without BSA, after four days of growth. The four asterisks on the top of the boxes indicate significant (*P* < 0.0001) differences compared to TemeculaL according to the two-tailed Student’s *t*-test. Measurements were repeated three times independently with at least 12 technical repeats each.
(TIFF)

S5 Fig Amino acid sequences alignment of PilQ (A), FimT3 (B), and PilT (C) across 133 *X. fastidiosa* strains. The phylogenetic tree was a midpoint-rooted maximum likelihood tree based on core genome alignment. Bootstrap confidence values for the branches were calculated from 1,000 replications and the phylogenetic distances are represented by a branch length of 0.01 substitutions per site. The columns besides the strain names infer competency (indicated by solid rectangles for competent strains and empty rectangles for non-competent strains) and contig numbers reflecting genome assembly completeness. Amino acid sequences were obtained using automlsa2 (v0.7.1; https://github.com/davised/automlsa2), which utilized BLASTp for sequence retrieval and MAFFT [51] for alignment. The phylogeny was visualized using iTOL [47].
(TIFF)

S6 Fig GWAS results using different tools and methods for identifying competence-associated genes. (A) Top subgraph with minimum *q* value generated by DBGWAS mapped to type IV pili genes *pilY1-1* and *pilY1-3*. (B) Top subgraph with minimum q value generated by DBGWAS mapped to the afimbrial adhesin PD0744 (*xadA2*).
(TIFF)

S7 Fig Pyseer results of different phenotype inputs: (A) log_10_ (Recombination rate*1e^8^), and (B) a scaled phenotype (0–3). Values of maximum –log_10_(P) in the y axis were plotted against average effect size in the x axis.
(TIFF)

S8 Fig Diagnostic Quantile-quantile (Q-Q) plots of GWAS analysis using Pyseer program which uses a mixed effect model. Q-Q plot shows the expected *p*-values versus observed p-values of each kmers using different phenotype inputs: (A) presence/absence, (B) log_10_ (Recombination rate*1e^8^), and (C) a scaled phenotype (0–3) based on recombination rate.
(TIFF)

S9 Fig Null distribution of treeWAS subsequent-test scores across all SNPs from 133 genomes. No SNP had a Bonferroni-adjusted *p*-value < 0.01.
(TIFF)

S1 Table Metadata of wildtype strains used for measuring natural competence in this study. (XLSX)

S2 Table Mutants and plasmids used in this study. (XLSX)

S3 Table Natural competence assay results with different types of donor DNA and recipient strains. (XLSX)

S4 Table Sequence identity of homologous regions in donor plasmid DNA against Temecula1 reference genome. (XLSX)

S5 Table Primers for generating PD0744 knockout mutant in TemeculaL. (XLSX)

S6 Table Ordinal scale recombination rate via natural competence (0–3). (XLSX)

S7 Table Significant unitigs in the top subgraph with the lowest *q*-value in DBGWAS results. (XLSX)

S8 Table Identified prophage candidates in Temecula1 genome. (XLSX)
