# Supplementary figures and images for: Natural competence in the bacterial pathogen Xylella fastidiosa varies across genotypes and is associated with adhesins
Source: PLoS Pathog. 2025 Dec 8;21(12):e1013757. doi: 10.1371/journal.ppat.1013757 (PMC12700405; doi:10.1371/journal.ppat.1013757)

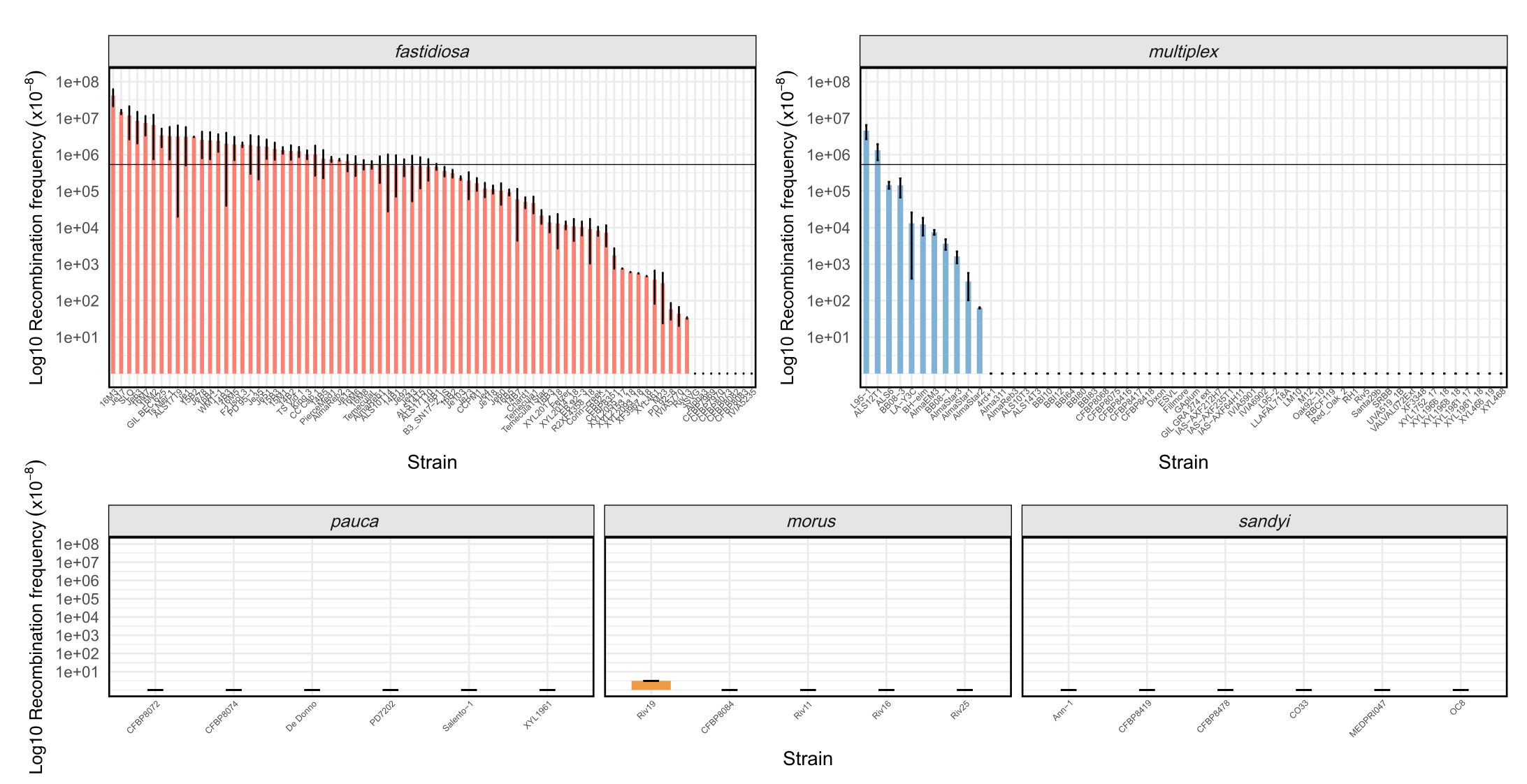

Supplement: S1 Fig — The recombination frequency was tested in vitro (see Materials and Methods) and data was log10-transformed. No bar indicates the recombination rate was below the detection limit and the strain was non-competent. Different bar colors indicate different subspecies. The black horizontal line indicates the average recombination rate of the reference strain TemeculaL. Experiments were repeated independently 1–3 times. Data represents means and standard errors. (TIFF) [file ppat.1013757.s001.tiff]

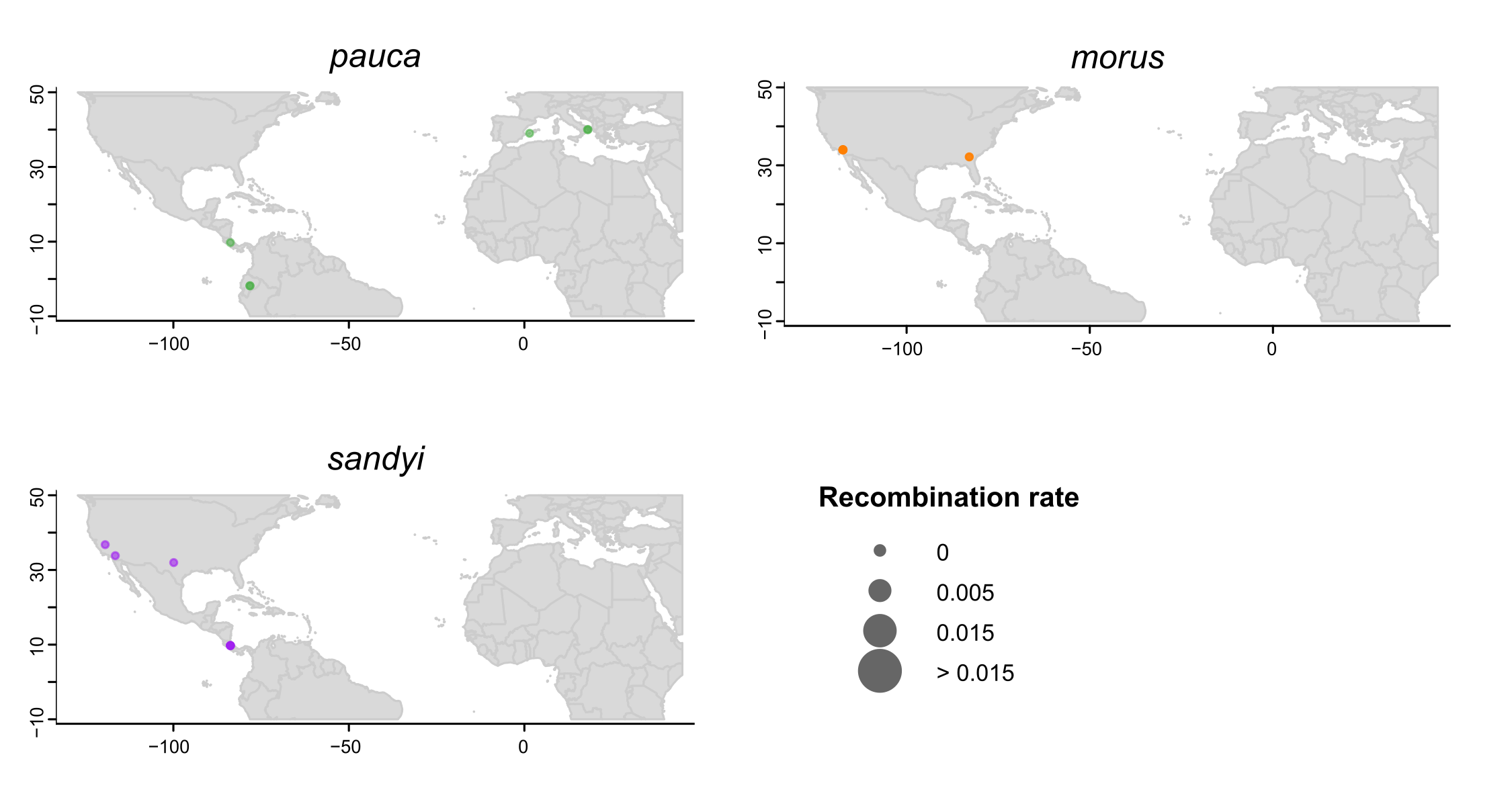

Supplement: S2 Fig — The dot size indicates range of recombination rates. Subsp. fastidiosa and multiplex are shown in Fig 2. The map was created using the R package ggplot2 [43]and maps. The base map layer (country and regional borders) comes from the maps package, which provides openly available map data derived from the CIA World DataBank II (https://www.evl.uic.edu/pape/data/WDB/). (TIFF) [file ppat.1013757.s002.tiff]

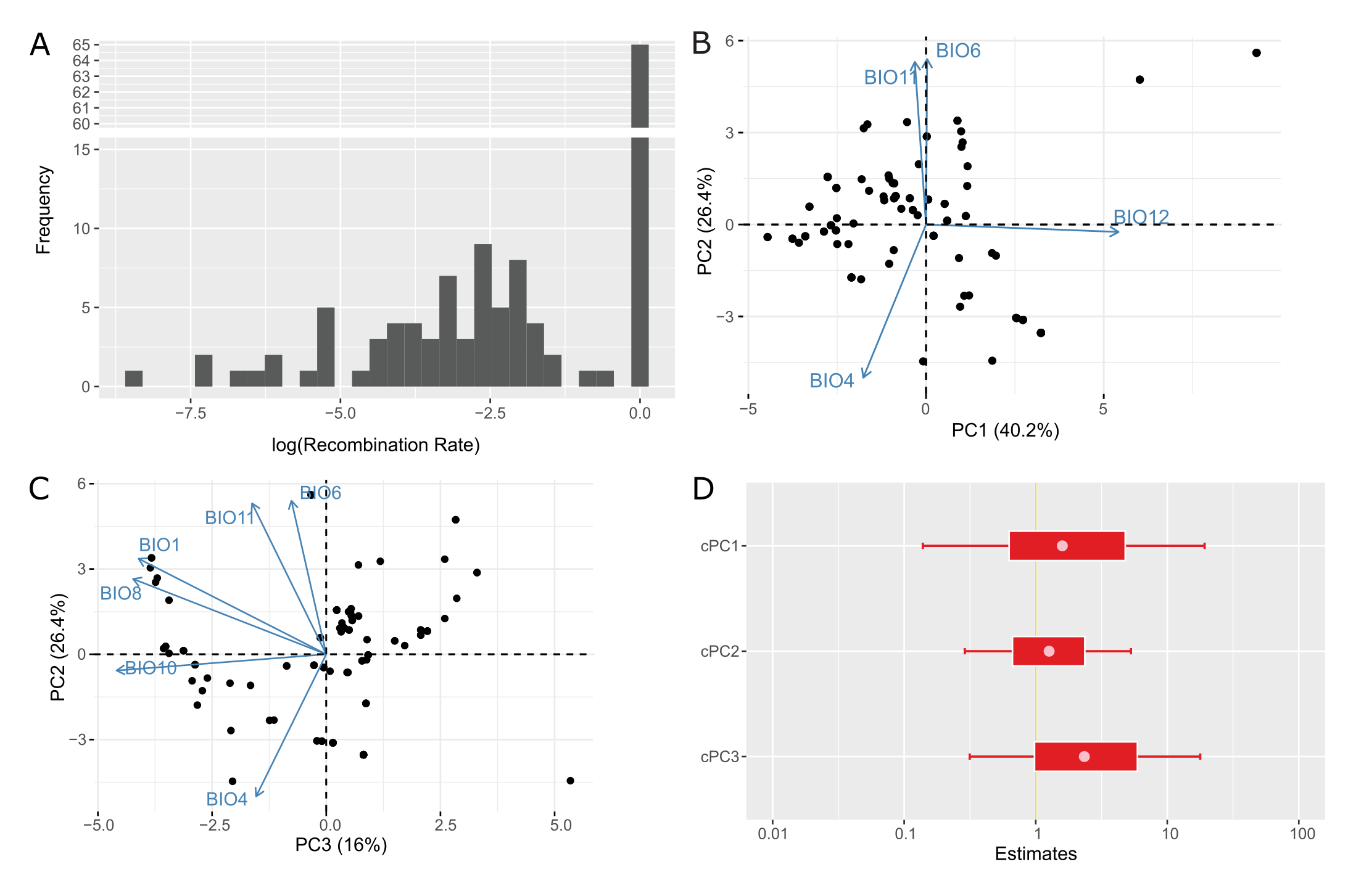

Supplement: S3 Fig — (A) Histogram representing the distribution of recombination rates in log scale. The distribution is heavily skewed, with a large number (n = 65) of “0” values. The y-axis is disjointed to accommodate the skewed distribution. (B, C) Principal Component Analysis (PCA) biplot of climatic variables showing the relationship between principal component pairs PC1 and PC2 (B)/ PC2 and PC3 (C) and variable contributions. Arrows represent variables with the greatest contributions: bio6 (minimum temp. of the coldest month), bio11 (mean temp. of the coldest quarter), bio12 (annual precipitation), bio4 (temp. seasonality), bio1 (annual mean temp.), bio8 (mean temp. of the wettest quarter), and bio10 (mean temp. of the warmest quarter). The length and direction of the arrows reflect the strength and direction of their correlation with the principal components. Only the labels of the top variables are displayed for clarity in each plot. (D) Estimated fixed effects for covariates on recombination rate in a generalized linear mixed-effect model. Boxplots indicate the 95% credible interval; all fixed effects were not significant, as they were all overlapping with 1. (TIFF) [file ppat.1013757.s003.tiff]

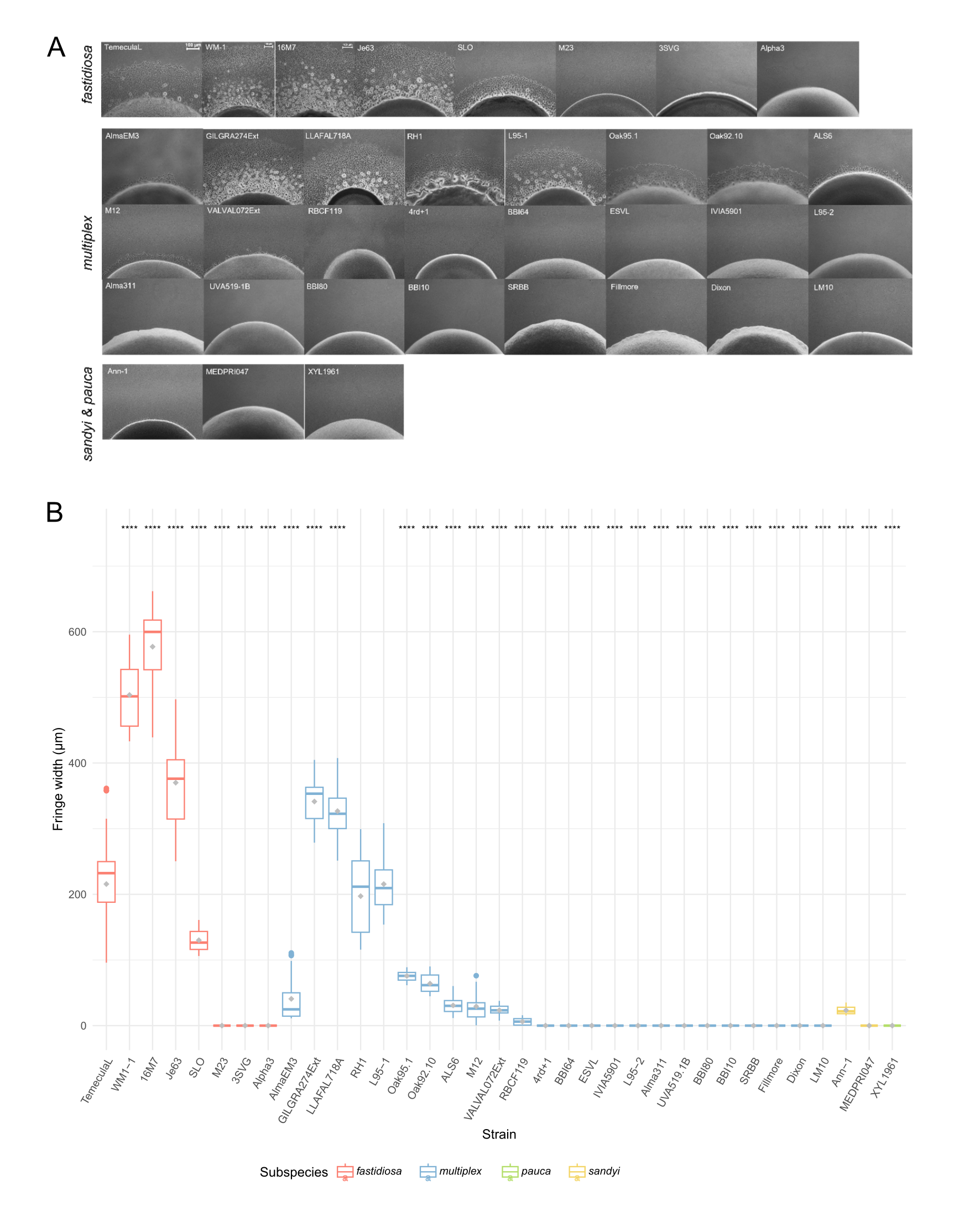

Supplement: S4 Fig — (A) Representative microscopic images of colony fringe of 35 tested X. fastidiosa strains grouped by subspecies. Images were captured at 10 × magnification. The scale bar on the first panel indicates 100 μm. (B) Quantitative twitching motility of the 35 X. fastidiosa strains, color-coded by subspecies. Twitching motility was determined by measuring fringe width of colonies spotted on PW plates without BSA, after four days of growth. The four asterisks on the top of the boxes indicate significant (P < 0.0001) differences compared to TemeculaL according to the two-tailed Student’s t-test. Measurements were repeated three times independently with at least 12 technical repeats each. (TIFF) [file ppat.1013757.s004.tiff]

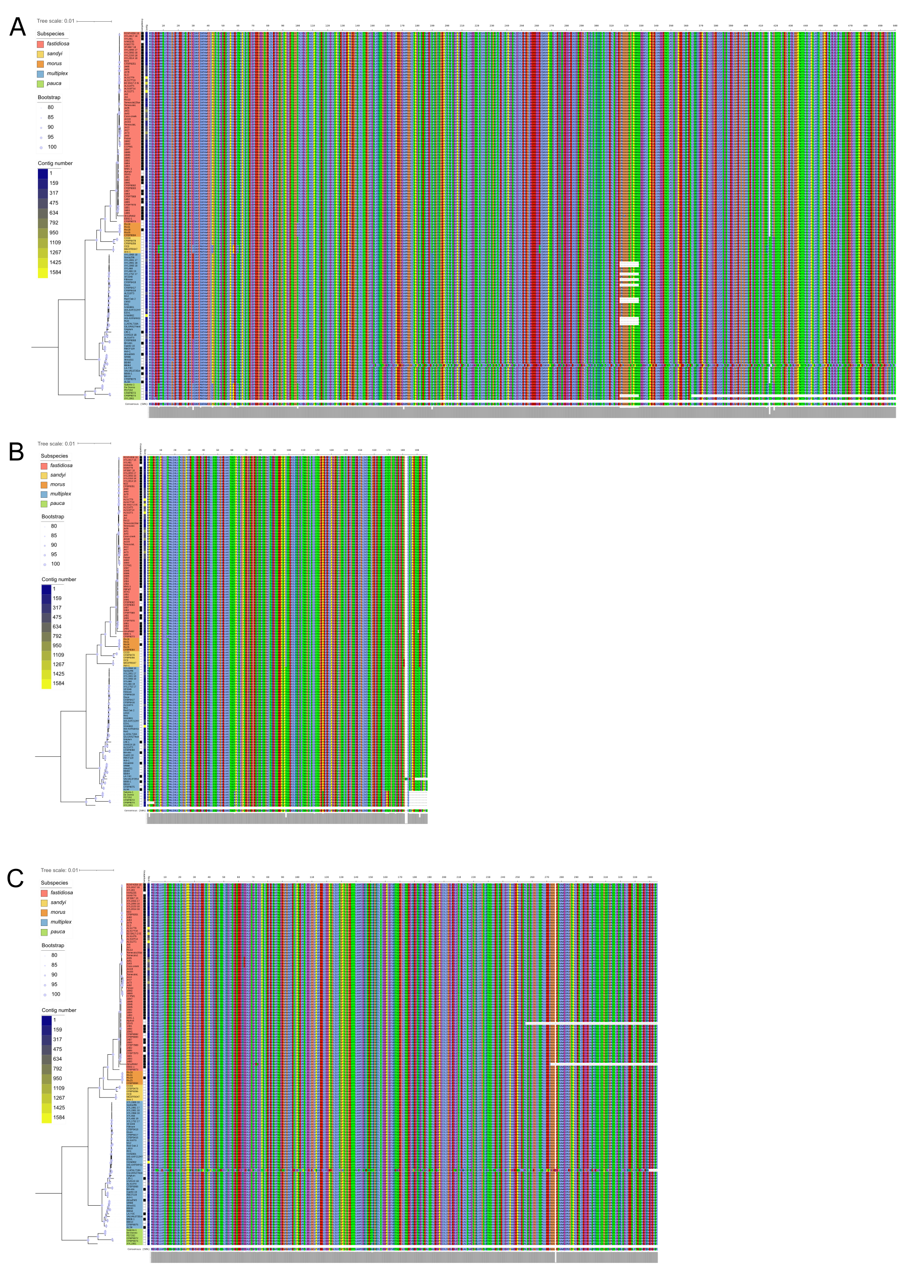

Supplement: S5 Fig — The phylogenetic tree was a midpoint-rooted maximum likelihood tree based on core genome alignment. Bootstrap confidence values for the branches were calculated from 1,000 replications and the phylogenetic distances are represented by a branch length of 0.01 substitutions per site. The columns besides the strain names infer competency (indicated by solid rectangles for competent strains and empty rectangles for non-competent strains) and contig numbers reflecting genome assembly completeness. Amino acid sequences were obtained using automlsa2 (v0.7.1; https://github.com/davised/automlsa2), which utilized BLASTp for sequence retrieval and MAFFT [51] for alignment. The phylogeny was visualized using iTOL [47]. (TIFF) [file ppat.1013757.s005.tiff]

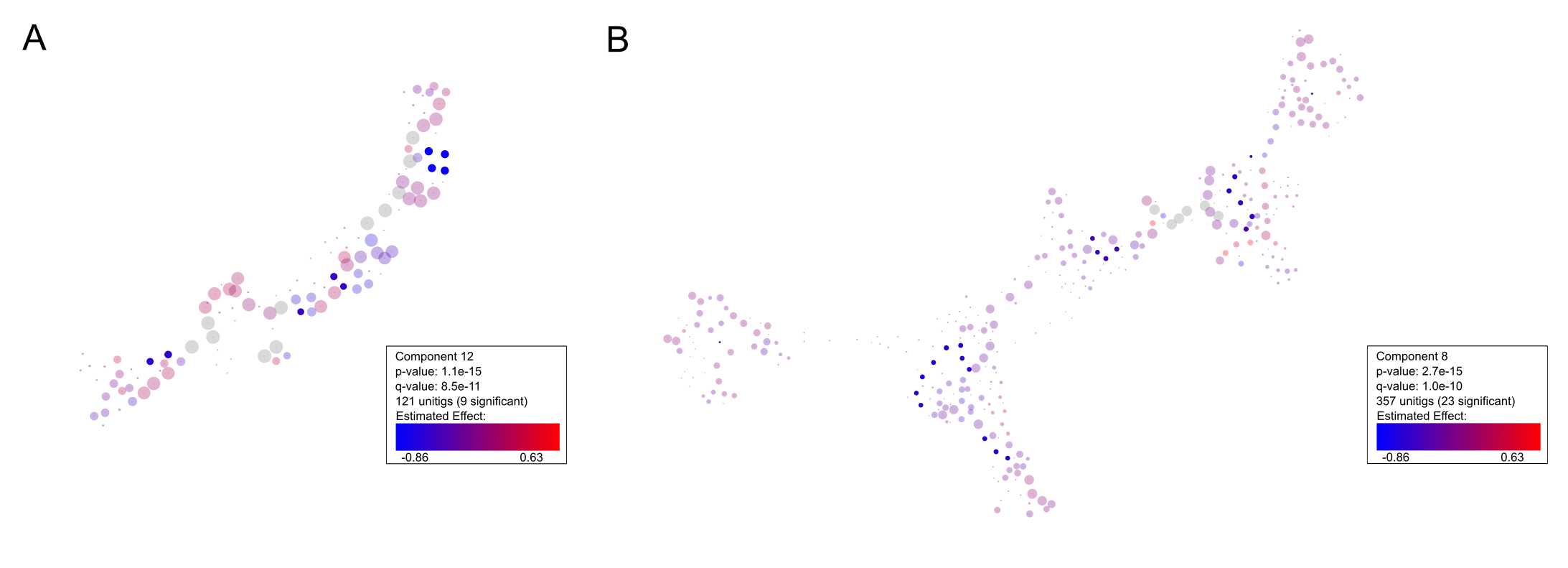

Supplement: S6 Fig — (A) Top subgraph with minimum q value generated by DBGWAS mapped to type IV pili genes pilY1-1 and pilY1-3. (B) Top subgraph with minimum q value generated by DBGWAS mapped to the afimbrial adhesin PD0744 (xadA2). (TIFF) [file ppat.1013757.s006.tiff]

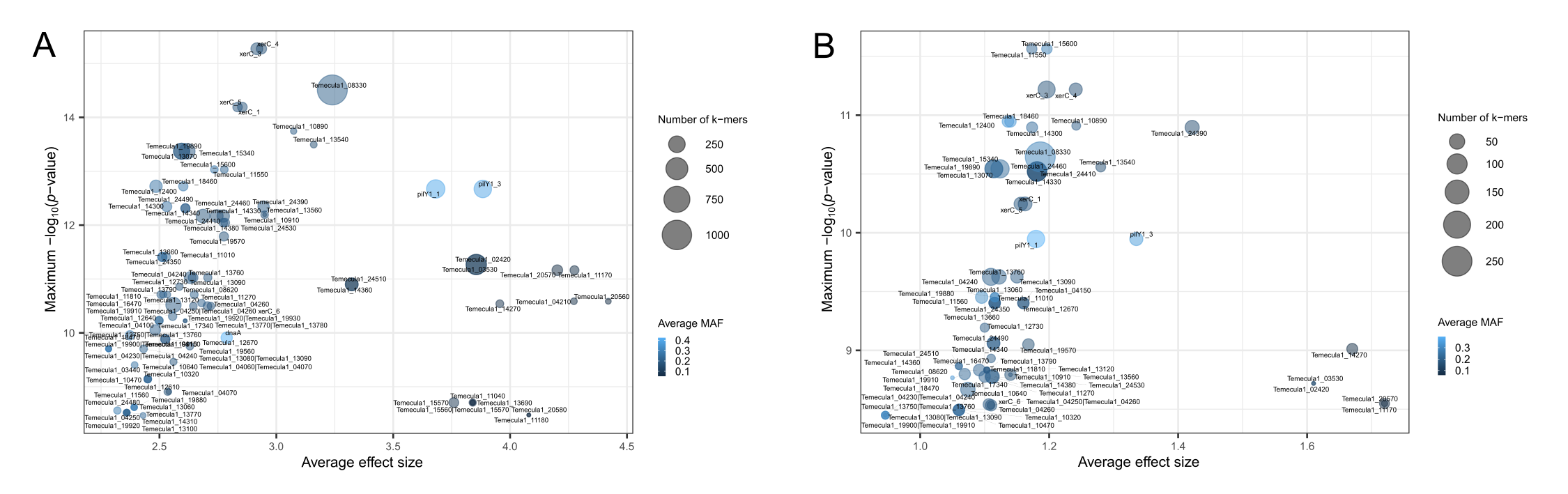

Supplement: S7 Fig — Values of maximum –log10(P) in the y axis were plotted against average effect size in the x axis. (TIFF) [file ppat.1013757.s007.tiff]

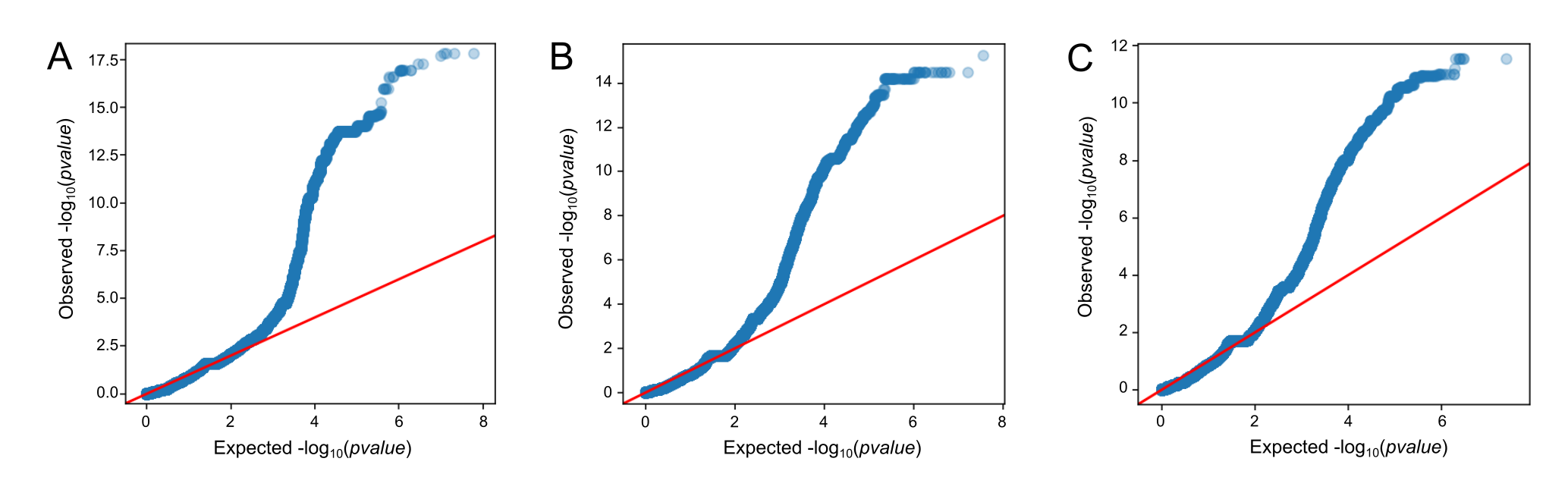

Supplement: S8 Fig — Q-Q plot shows the expected p-values versus observed p-values of each kmers using different phenotype inputs: (A) presence/absence, (B) log10 (Recombination rate*1e8), and (C) a scaled phenotype (0–3) based on recombination rate. (TIFF) [file ppat.1013757.s008.tiff]

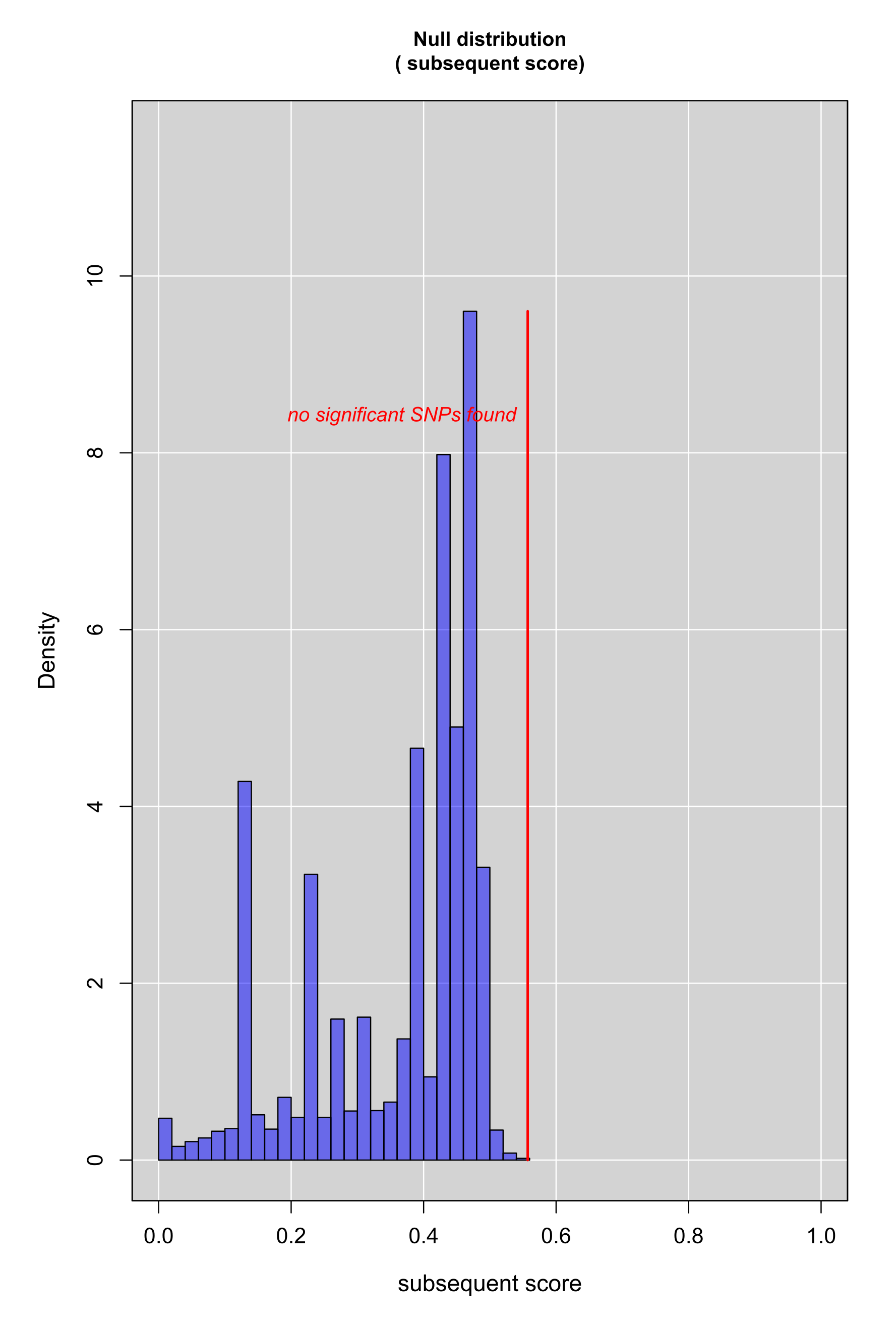

Supplement: S9 Fig — No SNP had a Bonferroni-adjusted p-value < 0.01. (TIFF) [file ppat.1013757.s009.tiff]
